# Ultrasonic Pulsating Water Jet Peening: Influence of Pressure and Pattern Strategy

**DOI:** 10.3390/ma14206019

**Published:** 2021-10-13

**Authors:** Gabriel Stolárik, Akash Nag, Jana Petrů, Jaroslava Svobodová, Sergej Hloch

**Affiliations:** 1 Faculty of Manufacturing Technologies, Technical University of Kosice, Prešov, Bayerova 1, 08001 Presov, Slovakia; stolarikg@gmail.com; 2 Department of Mechanical Engineering, Indian Institute of Technology (Indian School of Mines), Dhanbad 826004, India; akashnag1992@gmail.com; 3 Department of Mechanical Engineering, Vysoká Škola Báňská, Technical University, 708 00 Ostrava, Czech Republic; jana.petru@vsb.cz; 4 Faculty of Mechanical Engineering, University of J. E. Purkyně in Ústí nad Labem, 400 96 Ústí nad Labem, Czech Republic; jaroslava.svobodova@ujep.cz

**Keywords:** PWJ peening, surface modification, surface topography, microhardness

## Abstract

Peening techniques are nowadays attracting more research attention due to their association with the extending of the service life and improving surface texture of engineering components. Ultrasonic pulsating water jet peening represents a new way of mechanical surface treatment. Accelerated water droplets via hammer effect cause small elastic-plastic deformations on the surface. This work deals with peening of aluminum alloy using an ultrasonic pulsating water jet, where periodically acting water droplets were used as the peening medium. The aim of the work was the feasibility study of the peening process and to observe the effects of pressure (*p* = 10, 20 and 30 MPa) and pattern trajectory (linear hatch and cross hatch). The peened surfaces were analyzed by the surface roughness profile parameters Ra and Rz and the microhardness along the peening axis into the material. Graphically processed results show a clear increase of measured values with increasing pressure (*p* = 10, 20 and 30 MPa), where the roughness values ranged from 1.89 µm to 4.11 µm, and the microhardness values ranged from 43.3 HV_0.005_ to 47 HV_0.005_, as compared to 40.3 HV_0.005_ obtained for the untreated sample. The achieved results indicate potential using of an ultrasonic pulsating water jet as a new method of surface treatment of metals. By controlled distribution of water droplets, it is possible to achieve a local distribution of surface roughness, and at the same time, strengthening of the subsurface layers in the material without thermal influence on the material.

## 1. Introduction

In current technical practice, demands to keep functional components running, mainly in terms of their service life, are increasing day by day. By extending the service life of a part of a given machine, we can efficiently streamline the production process because of the less frequent requirement for repair or replacement of components due to wear [1]. Wear is mainly initiated on the surface of the component in the form of cracks or increased roughness of the material. Another negative factor affecting the service life of parts is the induced residual tensile stress caused by previous machining [2,3]. Some methods to overcome these shortcomings are already in practice, such as shot peening and laser peening [4]. However, these surface treatment methods cause some limitations, such as in the shot peening process, the induction of compressive residual stresses are limited to a certain depth. The shot peening process is undesirable for localized region treatment and a rougher surface finish is obtained due to interaction of the particles with the target material. For laser peening, a major limitation is the thermal effect, and specifically during treatment of materials with a lower melting point [5]. Therefore, it is currently necessary to find a suitable method of surface treatment where it would be possible to create an effective surface treatment without adverse effects. One of the possibilities is water jet (WJ) technology, where the material is removed or the surface is treated using a high-speed water jet that impacts the component [6]. This WJ technology can be used for various applications such as drilling, turning, cutting, cleaning, and strengthening [7]. The water jet peening method was first used in 1984 and subsequently went through various configurational improvements to enhance its ability. Surface treatment with continuous water jet (CWJ) is based on the principle of influencing the high-velocity water jet toward the target material with initial impact pressure acting for a shorter duration, followed by the stagnation pressure *p_s_*, which acts for the maximum duration of the impact and can be calculated using Equation (1) [8].

(1) $$p_{s}=\frac{1}{2}\rho v^{2}$$

With this method, it is possible to increase the hardness of the material by up to 15% by creating compressive stress in the surface layers of the material [9]. The supply pressure and standoff distance of the nozzle from the material surface were found to be the most significant parameters influencing the effect of the material. As the supply pressure was increased, the effect of strengthening also increased [10], whereas a lower standoff distance increased the surface roughness of the material [9]. Kunaporn et al. [11] verified the mathematical model for calculating and predicting the critical value of the standoff distance of the water nozzle from the workpiece. The study revealed that, if the actual distance used was less than a critical value, there was a significant improvement in the material properties; however, if this distance was greater than a critical value, then a significantly lower or no effect was observed on the material. It is, therefore, clear that the CWJ is significantly affected by the standoff distance, because air resistance enters the process and, thus, rapidly reduces the efficiency of the process [12].

Abrasive water jet (AWJ) technology is an improvement of CWJ and involves the impact of a high-velocity waterjet containing abrasive particles upon the surface of the material. This leads to the creation of small surface irregularities that alter the surface characteristics [13]. These changes in roughness and strength are significantly influenced by the abrasive grain size and the supply pressure [14]. Upon increasing the supply pressure and abrasive grain size, the roughness of the material increases [15]. When compared with CWJ, it is possible to increase the strength of the material by up to 25% using AWJ technology. However, the abrasive grains are pushed into the surface of the material, due to the kinetic energy of particles, which could cause problems in further machining of the workpiece or a change in operating properties [16]. This effect of increasing hardness was also confirmed in an experiment performed by Balaji and Jeyapoovan, where they observed an increase in hardness of up to 35.9% along with an increase in material roughness of 26.6% on the surface of AA 6063-T6 alloy. In this case, the pushing of fine abrasive particles into the material surface treated by AWJ was observed [17]. Other disadvantages of AWJ surface treatment include breakage or deformation of the abrasive grain upon initial impact on the material, which causes a significant reduction in the peening effect on the material when the same granulate is reused. As the number of uses of the same granulate increases, the degradation efficiency can drop abruptly by up to 4% per use [18].

Surface treatment with a pulsating water jet (PWJ) is a possible solution to the problems of embedding particles into the material (AWJ) and the need for a high-pressure water supply (WJ). This method of surface treatment does not use abrasive particles but instead features the repetitive impact of water clusters on the material. These shock waves of water agglomerate elongate the initial effect of water hammer, i.e., impact pressures *p_i_* (Equation (2)). This results in achieving higher material removal efficiency with even lower supply pressure (*p* < 40 MPa) than in the case of CWJ.

(2) $$p_{i}=\frac{v\rho_{1}c_{1}\rho_{2}c_{2}}{\rho_{1}c_{1}+\rho_{2}c_{2}}$$

The breakdown of continuous jet into pulsating jet can be achieved by various methods using rotating disks, self-resonating nozzles, Helmholtz resonating chambers, etc. However, in the current study, the PWJ is generated by piezoelectric ceramics that oscillate due to signals generated from the acoustic generator. These piezoelectric ceramics are mechanically attached to a vibrating cylindrical sonotrode, which causes a specific variation in the high-pressure water stream inside the acoustic chamber, whose length can be mechanically adjusted [19]. The high-pressure water fluctuations pass through the nozzle exit, where they are converted into a high-velocity modulated stream; then, at a certain distance from the exit, discrete bunches of water elements begin to form, which subsequently impact the material [20]. The distance required for the generation of discrete clusters of water elements is very important for the full utilization of the hammer effect [21]. When a high-pressure water jet (100 MPa or more) is used, this distance required for the formation of water agglomerates is rapidly increased and, thus, there is not much of an effect on the material [22]. Therefore, the use of lower pressure results in better efficiency during material treatment [23]; simultaneously, low-pressure generation does not require the installation of larger pumps, leading to economic savings. It is also very important to select the appropriate type of nozzle, as the effect on the material also differs significantly with different types of nozzle geometry. Srivastava et al. observed the effect of PWJ on the surface of the material using a circular and flat nozzle, where the results concluded that the circular nozzle induced residual compressive stresses to a much greater extent than a flat nozzle under the same conditions. This difference in effect is mainly due to the focusing of the water stream on a certain area. Therefore, it is clear that, when using a flat nozzle, the water jet is focused on a wider area compared to the circular nozzle, which leads to an overall lower peening effect on the surface of the material [24]. In terms of surface treatment, the study by Klich et al. dealt with the influence of PWJ on the surface of AW 5083 with different technological history (rough milling, fine milling, rolling, and planing). A significant influence of PWJ on the material was observed in the case of samples that were rolled. The highest increase in roughness ranged up to 150 times higher than the original roughness of the untreated PWJ material. The feed rate also had a very significant effect on the roughness of the material. The speeds tested were 0.5, 0.75, 1, 2, and 4 mm/s. The highest roughness was measured at the lowest feed rate; on the contrary, at the highest feed rate, the roughness did not change much for all samples [25].

Table 1 shows the parameters of the experiments performed using WJ, AWJ, and PWJ technology for the surface treatment of materials, where an effect was observed under certain changing parametric conditions.

It follows that PWJ technology can be expected to be used in industry for the surface treatment of materials, such as strengthening or cleaning purposes. The potential elimination of the shortcomings observed with CWJ and AWJ is possible using the PWJ method, which uses the impact of water agglomerates on the material and, thus, can affect the surface with approximately the same efficiency but at much lower operating pressures. The need for a lower operating pressure is a very important parameter, especially from an economic point of view, where the trend of future technologies is aimed at reducing input costs with increasing overall efficiency [27]. At the same time, discrete clusters of water are used without the abrasive that needs to be replaced, which is also significantly reflected in the advantages of this technology and the motivation to constantly improve it.

Another aspect during surface peening application which also requires consideration is the selection of optimal jet trajectory pattern. This parameter also plays a significant role in influencing the material surface when treating an entire given area. Also, the change in the trajectory pattern can also influence the jet impact, which finally affects the resultant material surface properties. In most cases, linear hatching pattern of jet trajectory is used, where the impacting jet acts on the surface only in one direction with a specific distance between two consecutive hatch lines. An example of such hatching is given in the work by Balaji D. S. and Jeyapoovan T. [17], where they monitored the effect of AWJ on AL 6063 and found that, with linear hatching, it is possible to increase the roughness and hardness by 26.6% and 35.9%, respectively. Another type of jet trajectory pattern is cross-hatching, which consists of linear hatching in one direction and an overlapping linear hatching in a direction perpendicular to the initial hatching with the same input conditions. This composite hatching was used in D. Arola’s [10] work on the surface of titanium steel (cpTi) and titanium alloy (Ti6A14V) using WJ and AWJ, where they monitored the effect of variation in the input conditions such as pressure and grain size (for AWJ) on the surface roughness of the material and residual compressive stress. It was observed that an increasing trend of roughness with increasing working pressure was obtained for both WJ and AWJ. Hlavacek P. [28] also used a cross-hatching approach with PWJ to erode a given area of concrete samples. The influence of number of cross-hatching cycles and the angle of impact of the PWJ on the maximal erosion depth and volumetric erosion rate was studied. Results showed that a 90° impact angle and a higher number of cycles generated higher erosion depth.

Limited works related to the comparison of the effect of trajectory patterns on surface treatment application were available for WJ technologies in the open literature. Moreover, studies that have looked at PWJ peening do not currently provide enough information to fine-tune the process for the desired outcome. It also does not provide sufficient information about PWJ peening of a given surface area with appropriate technological parameters and jet trajectory patterns, which limits its utilization in real industrial applications. 

This experiment tested the effect of PWJ peening on the surface integrity of an aluminum alloy using pressures (*p* = 10, 20, and 30 MPa), along with variation in the traverse speed of the PWJ head, which determines the frequency of water droplets impacting the material. Two types of pattern trajectories (linear and cross hatch) and their effects on the surface integrity during PWJ surface treatment of the material were used. This preliminary study opens a new avenue in utilizing water droplets at relatively low pressures compared to continuous abrasive water jets. The attributes define technological modification ultrasonic pulsating water jet as a suitable economic-environmental alternative to conventional water jet technologies.

## 2. Experimental and Methods

In this experiment, the effect of PWJ on the surface topography modification of aluminum alloy EN AW-1050A was studied. The material was selected due to its good mechanical properties, tensile strength of 145 MPa and modulus of elasticity of 69 GPa (provided by the manufacturer), microhardness of 40.3 HV_0.005_ and initial surface roughness of Ra 0.852 µm and Rz of 3.24 µm. Also, the chemical composition of the alloy was analyzed by X-ray spectrometer (PHI VersaProbe III, Physical Electronics Inc., Chanhassen, MN, USA) and is mentioned in Table 2.

**Figure 1:**
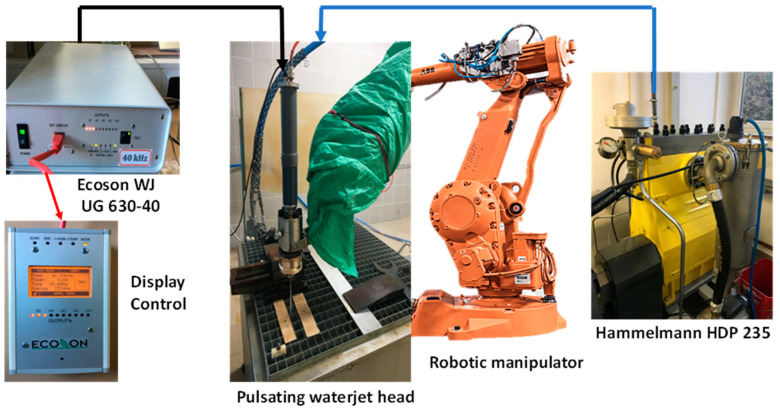
Overview of experimental setup for ultrasonic pulsating water jet composed from ultrasonic generator feeding the converter utilizing the inverse piezoelectric effect, causing the mechanical oscillation of the sonotrode in acoustic chamber.

This study also allows a detailed investigation to observe the changes in the material properties due to the variation in technological parameters. The equipment used in the experiment is shown in Figure 1.

The water pressure was generated using a Hammelmann HDP 253 high-pressure pump (Hammelmann, Oelde, Germany) with a maximum operating pressure of 160 MPa and a maximum flow rate of 67 L/min. The electrical impulses were generated using an Ecoson WJ UG 630-40 ultrasonic generator (Ecoson, Nove Mesto and Vahom, Slovakia) that caused the excitation of the piezoelectric crystals connected to the vibrating ultrasonic sonotrode. The ABB IRB 6640-180 robotic arm (ABB, Zürich, Switzerland) was used for controlling the movement of the PWJ head. The technological conditions used to perform the experiment are shown in Table 3.

**Figure 2:**
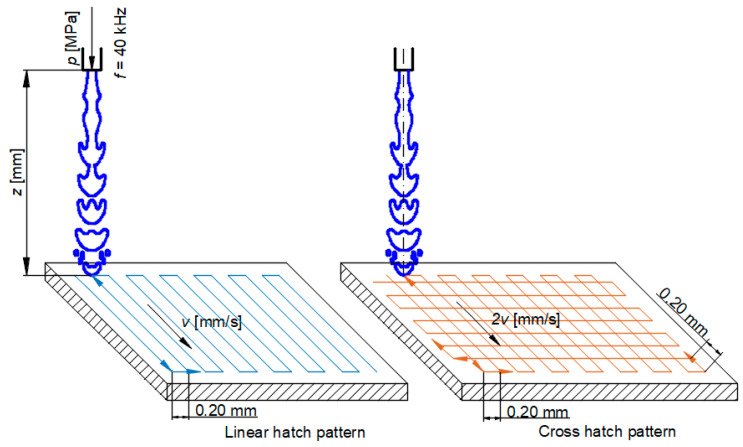
Different trajectory patterns used during the experiments for peening application.

The water pressure was selected to be 10, 20, and 30 MPa in order to sufficiently create the water hammer effect described above. Subsequently, a suitable acoustic chamber length was set for each experimental run depending upon the hydraulic parameters. The most suitable length of the acoustic chamber is one in which the acoustic generator works in the impedance region and simultaneously oscillates at the resonant frequency. This leads to the most efficient conversion of the electrical harmonic signal to the mechanical movement. In practice, this means achieving the lowest value of the working frequency with the highest possible output power. After determining the most suitable length of the acoustic chamber, an erosion test was conducted. This pilot test was carried out to determine the optimal standoff distance between the PWJ nozzle and the workpiece material. The standoff distance corresponding to the deepest generated groove was selected for the main experiments. With the selected water pressure, acoustic chamber length, and standoff distance, specific tests were performed on the aluminum alloy AW-6060 while changing the traverse speed and trajectory of the PWJ head (Figure 2). The first trajectory, i.e., a linear hatch pattern, was performed at 10 MPa with *v* = 10, 15, and 20 mm/s, followed by the second trajectory, i.e., a cross hatch pattern with *v* = 20, 30, and 40 m/s. The traverse velocity for the cross hatch pattern was always kept at double that used for the linear hatch pattern to maintain an identical peening or exposure time. This also resulted in an identical number of impacts/mm on the entire surface. A circular Hammelmann nozzle insert of 1 mm diameter, with discharge coefficient Cd = 0.92, was used for the experiments to focus the water jet on a confined area rather than the flat nozzle commonly used for peening purposes [24]. After performing the experiments, samples for further topographical and surface integrity investigations were selected on the basis of the surface roughness of the samples lying within the desired range with no sign of material erosion (Table 4). The samples for each supply pressure *p* = 10, 20, and 30 MPa, which were selected for the further investigations (Table 4), were repeated 3 times, keeping the technological parameters the same to ensure statistical unbiasedness and also to check the repeatability of the process.

**Figure 3:**
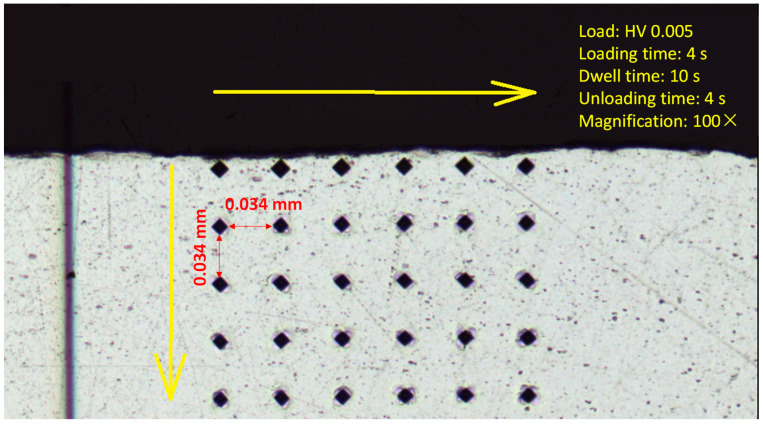
Microhardness measurement scheme under the peened surface and along material depth.

The surface roughness of the material was measured using a noncontact optical method. The peened surface was scanned using a MicroProf optical white light profilometer (FRT GmbH, Gladbach, Germany). The scanned data were imported into SPIP software to measure the surface roughness (Ra and Rz) of the peened surface. It is inevitable to point out that, besides complexity and presence of a number of stochastic effects in the PWJ technology characterization of roughness profile parameter Ra, i.e., only altitudinal characterization is not sufficient to evaluate the surfaces generated by this technology. In fact, two identical surfaces may exist with the same Ra yet from the point of view of Rz and, thus, regarding potential functionality, they are different. An investigation area of 5 mm × 5 mm was selected within the peened area with roughness parameters kept as follows for all the selected samples: cutoff wavelength = 0.8 mm; *n* of cutoffs = 4. The microhardness of the samples was measured using a micro-Vickers hardness testing machine, HM-220 B (Make: Mitutoyo). A load of 5 g was applied for indentation. The loading and unloading times were set to 4 s, and the holding or dwell time was maintained at 10 s. Five repetitions at the same distance from the peened surface were carried out. The indents were made at a consecutive distance of 50 µm from the previous indent. An array of indents was made, where the distance between any two indents in the same row or column was restricted to 0.034 µm from the corner of the indents. The microhardness values were recorded until a 950 µm depth from the peened surface. The scheme of microhardness testing is shown in Figure 3. A total of 120 measurements were taken for each sample for tracing the effect of PWJ peening below the sample surface.

## 3. Results and Discussion

**Figure 4:**
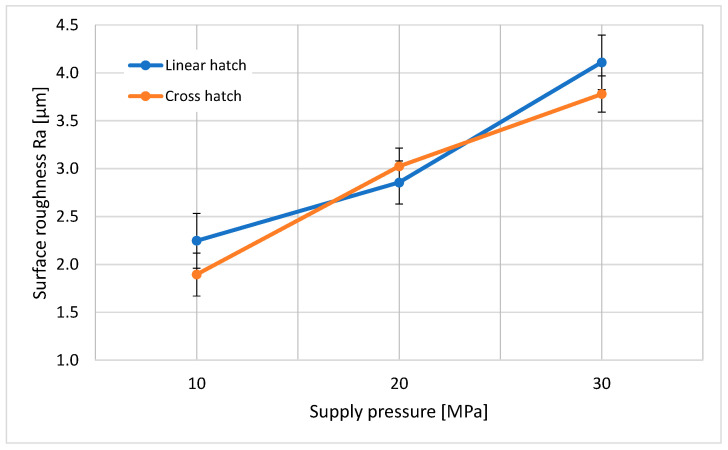
Influence of supply pressure *p* = 10, 20, and 30 MPa with linear and cross hatching on the surface roughness (Ra) of the target material affected by the pulsating water jet.

**Figure 5:**
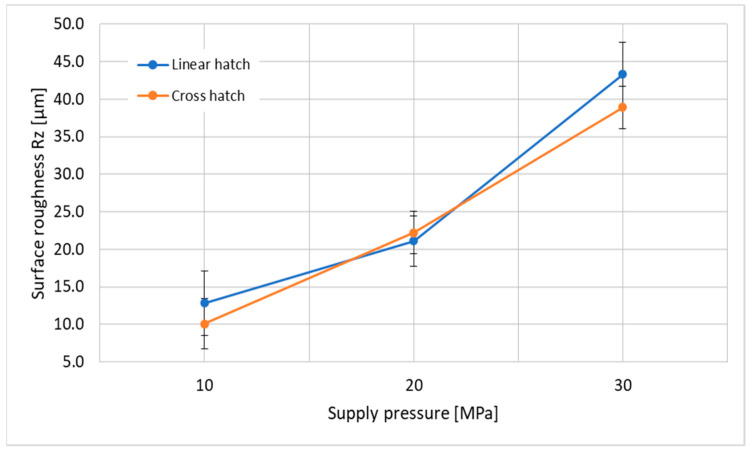
Influence of supply pressure *p* = 10, 20, and 30 MPa with linear and cross hatching on the surface roughness (Rz) of the target material affected by the pulsating water jet.

Figure 4 and Figure 5 show the measured surface roughness (Ra and Rz) generated by the PWJ under the different technological parameters shown in Table 4. A surface area of 5 mm× 5 mm was taken to measure the surface roughness values for each experimental run. The trend shows that, with an increase in the supply pressure, the Ra and Rz values increased. This can be attributed to the higher flow rate and hydraulic energy of the jet at *p* = 30 MPa (*Q* = 10.4 L/min, *P* = 5.2 kW) compared to *p* = 10 MPa (*Q* = 6 L/min, *P* = 1 kW). With an increase in the hydraulic energy, the tendency of the jet to cause measurable erosion in the form of surface roughness also increased. Therefore, it can be noted that a maximum mean surface roughness of Ra = 4.11 µm and Rz = 43.3 µm at *p* = 30 MPa was measured as compared to Ra = 2.247 µm and Rz = 12.84 µm at *p* = 10 MPa with a linear hatch pattern. The effect of the number of passes and the traverse speed of the jet can also be observed from the graph. The traverse speed was kept such that it took the same amount of peening time to complete the entire cross hatch pattern and linear hatch pattern. It can be noted that Ra values of 1.894 µm and 2.247 µm and Rz values of 10.10 µm and 12.84 µm were measured for the cross hatch pattern with *v* = 40 mm/s and linear hatch pattern with *v* = 20 mm/s, respectively. For the linear hatch pattern with *v* = 20 mm/s, the number of impacts per unit length of the material could be approximated to 2000 impacts/mm. On the other hand, for the cross hatch pattern with *v* = 40 mm/s, 1000 impacts/mm were induced into the material twice, but with a time interval between both sets of impacts. This allowed the material to relax and deaccelerate the erosion rate as compared to the linear hatch pattern. Therefore, the material surface treated using the cross hatch pattern showed a lower surface roughness compared to the linear hatch pattern (Ra = 4.11 µm, Rz = 43.3 µm and 3.78 µm, Rz = 38.9 µm for the linear hatch pattern and cross hatch pattern, respectively, at *p* = 30 MPa). However, at *p* = 20 MPa, this trend was the opposite (Ra = 2.855 µm, Rz = 21.10 µm and 3.024 µm, Rz = 22.21 µm for the linear hatch pattern and cross hatch pattern, respectively), which may have been due to reasons such as local material properties, instability in the motion of the jet, and improper modulation of the jet.

**Figure 6:**
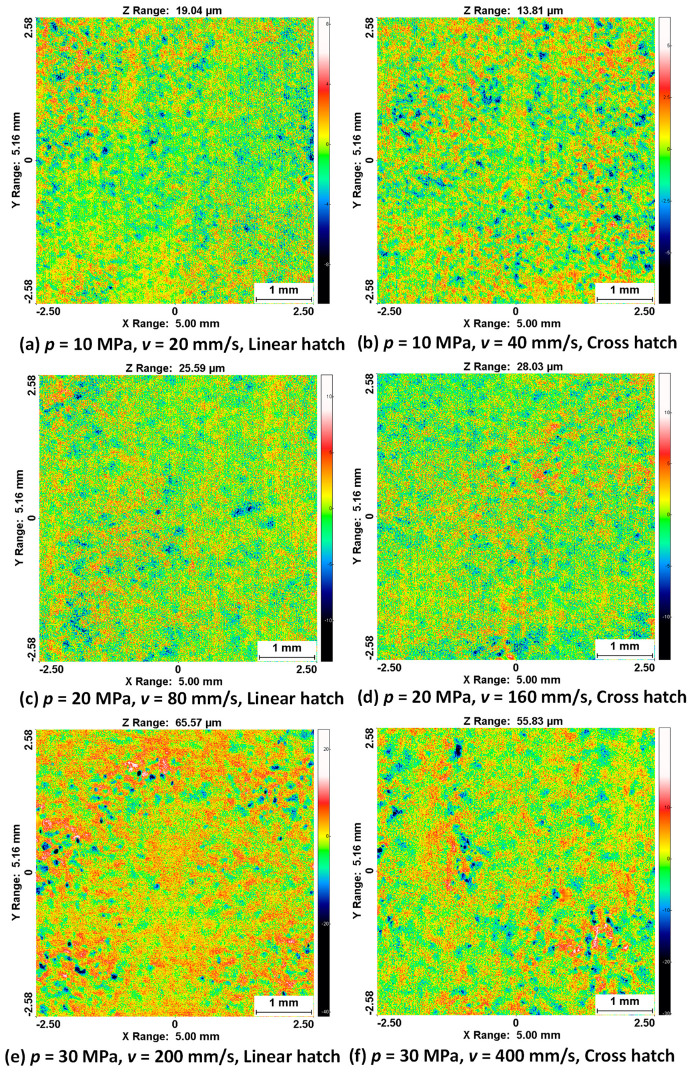
Surface topography of the surfaces generated after pulsating water jet treatment using different technological settings (area of interest: 5 × 5.16 mm).

The 2D surface topography of the resultant surface in Figure 6 shows the trend of increasing roughness with increasing pressure, as mentioned above. In this figure, the distance between the highest peak of the measured area to the lowest valley is presented. The surface generated during the experimental run with an operating pressure *p* = 10 MPa showed a higher unevenness with the linear hatch pattern compared to that with the cross hatch pattern; the difference in peak-to-valley value was measured as 5.23 µm. A higher surface roughness of the sample was measured with the linear hatch pattern (Ra = 19.04 µm) with *v* = 20 mm/s compared to the cross hatch pattern (Ra = 13.81 µm) with *v* = 40 mm/s. The experimental displacement of the jet between two consecutive lines was restricted to 0.2 mm, and the nozzle diameter used for experiments was *d* = 1.0 mm (Figure 2). This means that the whole central part of the material surface was impacted by the PWJ five times. This action led to stressing the sample surface only in one direction, which caused a significant weakening of the surface layer of the material in the longitudinal direction. On the contrary, in the cross hatch pattern, the material was impacted by the PWJ in both the longitudinal and the transverse direction. The traverse speed used during this experimental run was double (*v* = 40 mm/s) that for the linear hatch pattern (*v* = 20 mm/s), to maintain an identical treatment period for both treatment patterns. Therefore, the number of impacts per unit length of the material and the interaction time between the PWJ and the material surface was reduced to half at any point in time, resulting in a lower erosive effect. However, during the next phase of the treatment, i.e., along the traverse direction, the surface was again impacted with 1000 impacts per unit length, which altered the initial roughness generated along the longitudinal direction of the surface and made the overall sample surface more uniform. The trend of higher surface roughness after PWJ treatment was also observed at *p* = 30 MPa, where the linear hatch pattern resulted in an increase of 9.74 µm in peak-to-valley value. The peak-to-valley value was measured at 65.57 µm and 55.83 µm for the linear and cross hatch patterns with traverse speeds of *v* = 200 and 400 mm/s, respectively. The reasons for the higher surface roughness during the linear pattern trajectory were similar to those described for supply pressure *p* = 10 MPa.

An interesting deviation in material roughness was observed for the samples generated using *p* = 20 MPa, with traverse speeds *v* = 80 and 160 mm/s for the linear and cross hatch pattern trajectories. In this case, the values measured with the linear hatch pattern were lower compared to the cross hatch pattern, which was opposite to the previous two supply pressures *p* = 10 and 30 MPa. At traverse speed *v* = 80 mm/s with the linear hatch trajectory, a maximum peak-to-valley value of 25.59 µm was measured compared to the 28.03 µm measured at *v* = 160 mm/s with the cross hatch trajectory, generating a mutual difference of 2.44 µm. Interestingly, although the linear hatch pattern had a lower measured value, the overall difference between the linear and cross hatch pattern trajectories was the lowest among all three supply pressures *p* = 10, 20, and 30 MPa. Since the difference between these values was the smallest, various minor uncertainties in setting up the parameters used in the experiment, including the setting of the nozzle distance from the workpiece, variation in the flow rate of the water inlet, or local material properties, may have led to the degradation of the surface during the cross hatch experiment.

**Figure 7:**
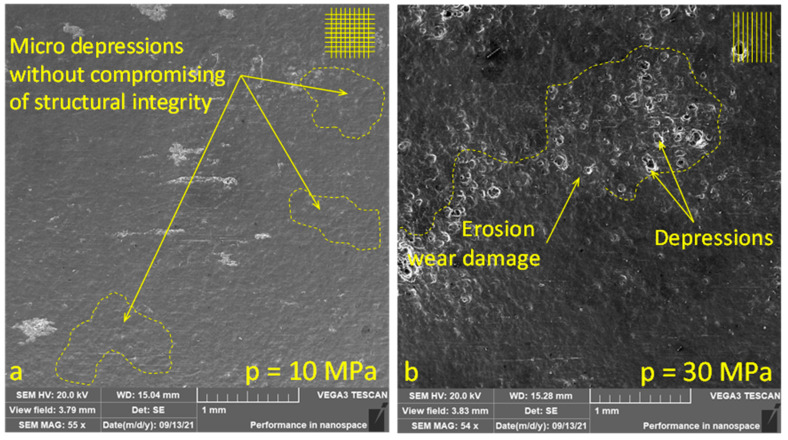
Sample surfaces with microstructures caused due to droplet erosion, obtained by SEM performed at (**a**) *p* = 10 MPa, with cross hatching resulted in subsurface microhardness of 44.0 ± 2.84 HV; (**b**) *p* = 30 MPa with linear hatch resulted in subsurface microhardness of 46.1 ± 2.98 HV.

The following Figure 7 shows a comparison of surfaces obtained by SEM (Tescan, Brno, Czech Republic) treated at a pressure of 10 MPa (Figure 7a) and 30 MPa (Figure 7b). The surface at a pressure of 10 MPa was treated in a cross hatch pattern trajectory (Figure 7a). The treated surface was obtained without compromising the structural integrity of the material surface. The hardening values obtained by measuring the microhardness are in the range of 44.0 ± 2.84 HV_0.005_. Slightly higher hardness values of 46.1 ± 2.98 HV_0.005_ were obtained at a pressure of 30 MPa with a linear hatch pattern (Figure 7b). However, erosion damage wear can be clearly observed on the surface for samples treated with *p* = 30 MPa. Moreover, it must be noted that for materials with a different physical property, the optimal technical parameters to achieve desirable roughness values along with enhanced microhardness will differ. However, these results suggest the possibility that surfaces can be treated at low pressures, as suggested by some published studies [26,29].

**Figure 8:**
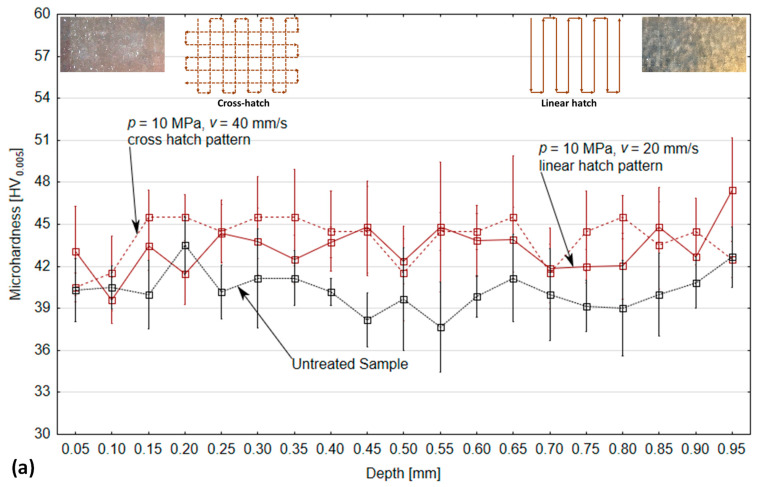

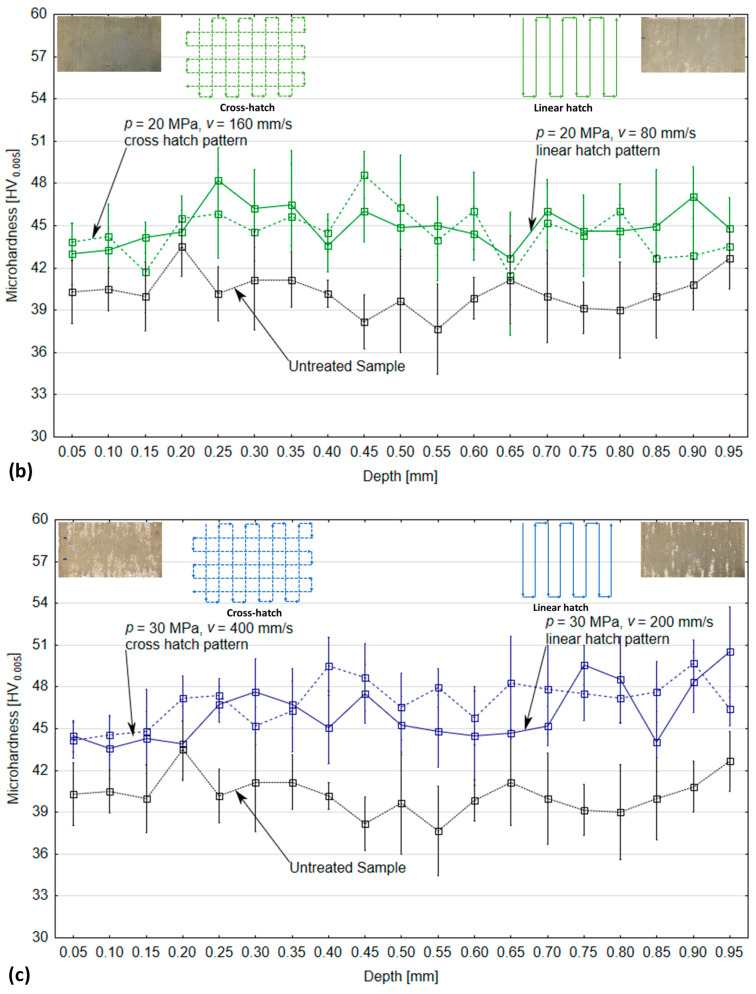
Variation in microhardness along the depth from the peened surface with (**a**) *p* = 10 MPa, (**b**) *p* = 20 MPa, and (**c**) *p* = 30 MPa for both linear and cross hatch pattern trajectories.

Figure 8a shows the trend of microhardness change after PWJ treatment with working conditions of *p* = 10 MPa. The graph shows that the microhardness values varied at each point throughout the range of measured depth. For a better understanding of the effect of PWJ on the microhardness of the material in this experiment, the hardness of the untreated material (40.3 HV_0.005_) was measured before the start of the experimental runs. In the graph, these values are marked in black. It can be observed that the microhardness increased considerably over the entire range of the measured depth, except for small deviations just near the surface, which were, however, not very significant. There was a nonlinear rise and fall in microhardness values across the entire measured range. The lowest microhardness was measured at depths of 0.10 and 0.05 mm below the surface, with values of 40.50 and 40.33 HV_0.005_ for the linear and cross hatch patterns, respectively. On the other hand, the highest microhardness was measured at depths of 0.95 and 0.15 mm below the surface with values of 47.46 and 45.5 HV_0.005_ for the linear and cross hatch patterns, respectively. When comparing the affected surface with the original unaffected surface, it can be seen that the microhardness increased with approximate deviations across the whole range. This is interesting because the pressure waves, after hitting the material, propagated at different speeds in different directions and lost their strength after a short distance, but the graph shows that the material was strengthened approximately equally from a depth of 0.15 to 0.95 mm.

At *p* = 20 and 30 MPa (Figure 8b,c), the microhardness trend was approximately similar to that at *p* = 10 MPa. A nonlinear change in microhardness at each measuring point can also be seen with increasing depth from the surface of the material. The lowest and highest microhardness values measured were 42.68, 41.48 HV_0.005_ and 48.2, 48.62 HV_0.005_ for the linear and cross hatch patterns, respectively, at *p* = 20 MPa. At *p* = 30 MPa, the lowest and highest microhardness values measured were 43.62, 44.16 HV_0.005_ and 50.52, 49.73 HV_0.005_ for the linear and cross hatch patterns, respectively. A comparison of the microhardness values, before and after the experiment, indicated a clear increase in both cases. In contrast to the pressure of 10 MPa, in these cases (*p* = 20 and 30 MPa), the microhardness increased across the whole measured depth range. At no point did the unaffected hardness curve intersect with the hardness curve after the experiment. This trend of higher microhardness was attributed mainly to an increase in operating pressure, which induced higher compressive stresses in the material, resulting in the generation of slip lines and a modification of the grain boundaries along with grain refinement.

However, from the above results, it is clear that the effect of PWJ on the surface microhardness did not have the same effect at different depths from the material surface, and the overall effect was significantly influenced by the supply pressure used. The type of pattern trajectory led to minimal changes in microhardness, because, at certain depths of measurement, the higher value of microhardness alternated for linear and cross hatch pattern samples.

**Figure 9:**
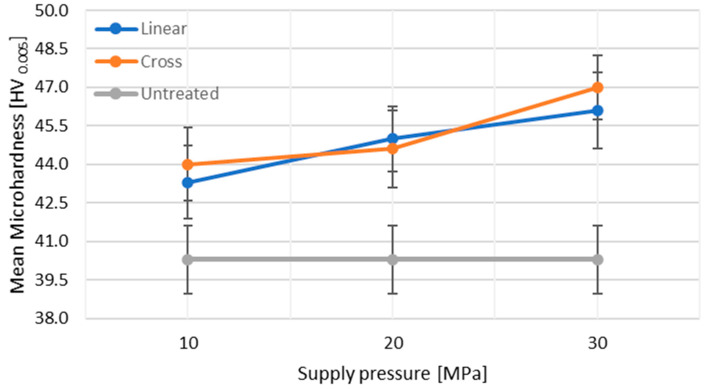
Variation in mean microhardness across the measured depth with different peening pattern and supply pressure.

Figure 9 shows the variation trend of microhardness after the impact of PWJ with respect to the trajectory pattern and the supply pressure. An overall comparison of the microhardness after treatment with that of the unaffected material showed an increase in the mean microhardness values with an increase in supply pressure. It can be observed that an increase of 16.62% in the mean hardness values as compared to the unaffected samples was measured at *p* = 30 MPa, respectively. This highlights the efficiency of the PWJ, i.e., even with lower supply pressure *p* = 30 MPa, a 16–17% increase in subsurface microhardness is observed as compared to 35.9% reported by Balaji, D.S. and Jeyapoovan, T. [17] when using AWJ with *p* = 160 MPa on aluminum alloy samples.

The increasing trend of microhardness was attributed to the induction of higher compressive stresses in the material, as discussed previously [30]. However, compared to the roughness trend, the microhardness trend showed the opposite dependency with the type of pattern trajectory. It can be observed that the cross hatch pattern showed higher values of mean hardness at *p* = 10 and 30 MPa as compared to the linear hatch pattern, which was in contrast to the trend for roughness values (Figure 4). At *p* = 20 MPa, the opposite trend was observed. This was attributed to the more uniform induction of compressive stress in the material, resulting in better grain refinement and material hardening. This also correlated with the lower roughness values obtained with the cross hatch pattern at *p* = 10 and 30 MPa. Therefore, it can be concluded that the proper selection of technological parameters such as supply pressure and traverse speed along with treatment pattern can make PWJ a competitive method for surface treatment applications.

## 4. Conclusions

In this experiment, the effect of PWJ on the surface topography of the material in terms of surface roughness and subsurface microhardness was observed. The experimental conditions were set such that it was possible to clearly compare the influence of the technological parameters and treatment strategy of the samples, as well as discuss their various effects. The pressures used were *p* = 10, 20, and 30 MPa, and the treatment pattern (linear and cross hatch) along with the appropriate traverse speed was changed for each pressure. The main observations of the experiments are listed below.

(1) The influence of PWJ on the surface roughness of the material (Figure 4) showed that, with increasing pressure, the roughness of the material also increased. However, it was observed that the lowest roughness value corresponded to the cross hatch treatment pattern at *p* = 10 MPa (1.89 µm) and, conversely, the highest roughness was measured with the linear hatch pattern at *p* = 30 MPa (4.11 µm).
(2) The same increasing trend was observed when measuring the peak-to-valley distance, where the difference between peaks and valleys also increased with increasing pressure.
(3) The treated samples, after measuring the microhardness, showed an increase in the subsurface microhardness with increasing working pressure. However, the microhardness (Figure 8a–c) of the samples varied nonlinearly and randomly along the depth for both linear and cross hatch pattern trajectories.
(4) The mean microhardness values (Figure 9) clearly showed an increase with increasing working pressure, as well as an effect of the treatment pattern used at fixed pressure. The increase in hardness values with the cross hatch pattern was due to an even distribution of the impacted stress across the whole surface as compared to the unidirectional effect induced with the linear hatch pattern. A maximum increase in microhardness of 16.62% was observed as compared to the unaffected material.
(5) The use of an ultrasonically excited pulsating water jet eliminates the use of abrasive particles, as is the case with abrasive water jets, which reduces the environmental and economic problems of the surface treatment process.
(6) PWJ forms a structure similar to microstructures for bone due to repeated droplet erosion. This knowledge will be used in further research in the surface treatment of titanium alloys for cemented and cementless orthopedic implants. This direction is also indicated by the published results in the work [31].

Therefore, from the above results, it can be noted that the working pressure had the greatest effect on the surface roughness and subsurface microhardness. Furthermore, the variation in treatment pattern had an effect on the resulting surface topography of the affected material. Overall, it can be concluded that PWJ can be used as an effective alternative for the peening of machine components and structures to enhance their service lifetime.

## Figures and Tables

**Table 1:** Related work.

Study	Method	Material	Experimental Conditions					Effect
			*p* (MPa)	f (kHz)	*d* (mm)	z (mm)	*v* (mm/s)	
[13]	Abrasive water jet (AWJ)	Ti6Al4V	80 140 210 280	-	0.3	150	63.5	Effective cleaning with suitable parameters; strengthening
[14]	AWJ	AISI 304 Ti6Al4V	103 172 262	-	0.36	152 203 254	17 25.3 33.8	Hardness increased by 25%
[10]	AWJ	cpTi Ti6Al4V	80 140 210 280	-	0.3	150	63.5	Increased pressure; decreased residual stress
	Continuous water jet (CWJ)		140 210 280					Increased pressure; increased residual stress
[9]	CWJ	Al 7075-T6		-	0.3	13–152		Increased compressive residual stresses by up to 15%
[11]	CWJ	Al7075-T6	103	-	0.3	24 36 47	12.7	Increased “z” from the mathematical calculation; reduced the fatigue resistance of the material
			207			36 59 83		
			310			44 77		
[24]	Pulsating water jet (PWJ)	AISI 304	20	20	1 (10°) Flat	30	0.25 1 1.5 2.5	Lower hardness; lower residual stress
					1.9	45		Higher hardness; higher residual stress
[22]	PWJ	AISI 304	40 60 80 100	20	1.19	15 23 31	5 15 25	Higher erosion effect at low pressures
[26]	PWJ	AISI 304	20	20	1.9	45	0.25	Revealed as a promising new method for the surface treatment of welds
							1	
							2	
			40			70	3	
							4	
			60			100	5	
							6	

**Table 2:** Elemental composition of EN AW–1050A used for the current study.

Element	Al	Si	Fe	Mg
Composition (wt.%)	99.42	0.24	0.34	Negligible

**Table 3:** Experimental conditions.

Pressure *p* (MPa)	Frequency *f* (kHz)	Chamber Length *L_c_* (mm)	Standoff Distance *z* (mm)	Speed *v* (mm/s)	Pattern
10	42.5	7	42	10, 15, 20	Linear hatch
				20, 30, 40	Cross hatch
20	42.5	7	43	40, 60, 80	Linear hatch
				80, 120, 160	Cross hatch
30	42.5	9	46	160, 200, 240	Linear hatch
				320, 400, 480	Cross hatch

**Table 4:** Samples selected for further measurement and investigation.

Pressure *p* (MPa)	Frequency *f* (kHz)	Chamber Length *L_c_* (mm)	Standoff Distance *z* (mm)	Speed *v* (mm/s)	Pattern
10	42.5	7	42	20	Linear hatch
				40	Cross hatch
20	42.5	7	43	80	Linear hatch
				160	Cross hatch
30	42.5	9	46	200	Linear hatch
				400	Cross hatch

## Data Availability

The data presented in the study are available on request from the corresponding author.
